# Classification of Widely and Rarely Expressed Genes with Recurrent Neural Network

**DOI:** 10.1016/j.csbj.2018.12.002

**Published:** 2018-12-14

**Authors:** Lei Chen, XiaoYong Pan, Yu-Hang Zhang, Min Liu, Tao Huang, Yu-Dong Cai

**Affiliations:** a School of Life Sciences, Shanghai University, Shanghai 200444, People's Republic of China; b College of Information Engineering, Shanghai Maritime University, Shanghai 201306, People's Republic of China; c Shanghai Key Laboratory of PMMP, East China Normal University, Shanghai 200241, People's Republic of China; d Department of Medical Informatics, Erasmus MC, Rotterdam, the Netherlands; e Institute of Health Sciences, Shanghai Institutes for Biological Sciences, Chinese Academy of Sciences, Shanghai 200031, People's Republic of China

**Keywords:** Widely expressed gene, Rarely expressed gene, Enrichment theory, Minimum redundancy maximum relevance, Incremental feature selection, Recurrent neural network

## Abstract

A tissue-specific gene expression shapes the formation of tissues, while gene expression changes reflect the immune response of the human body to environmental stimulations or pressure, particularly in disease conditions, such as cancers. A few genes are commonly expressed across tissues or various cancers, while others are not. To investigate the functional differences between widely and rarely expressed genes, we defined the genes that were expressed in 32 normal tissues/cancers (i.e., called widely expressed genes; FPKM >1 in all samples) and those that were not detected (i.e., called rarely expressed genes; FPKM <1 in all samples) based on the large gene expression data set provided by Uhlen et al. Each gene was encoded using the gene ontology (GO) and Kyoto Encyclopedia of Genes and Genomes (KEGG) enrichment scores. Minimum redundancy maximum relevance (mRMR) was used to measure and rank these features on the mRMR feature list. Thereafter, we applied the incremental feature selection method with a supervised classifier recurrent neural network (RNN) to select the discriminate features for classifying widely expressed genes from rarely expressed genes and construct an optimum RNN classifier. The Youden's indexes generated by the optimum RNN classifier and evaluated using a 10-fold cross validation were 0.739 for normal tissues and 0.639 for cancers. Furthermore, the underlying mechanisms of the key discriminate GO and KEGG features were analyzed. Results can facilitate the identification of the expression landscape of genes and elucidation of how gene expression shapes tissues and the microenvironment of cancers.

## Highlights

• Some genes are widely expressed across tissues or various cancers.
• A number of genes are rarely expressed across tissues or various cancers.
• The functional differences between widely and rarely expressed genes were studied.
• Several GO terms and KEGG pathways were extracted and analyzed.

## Graphical Abstract

**Figure:**
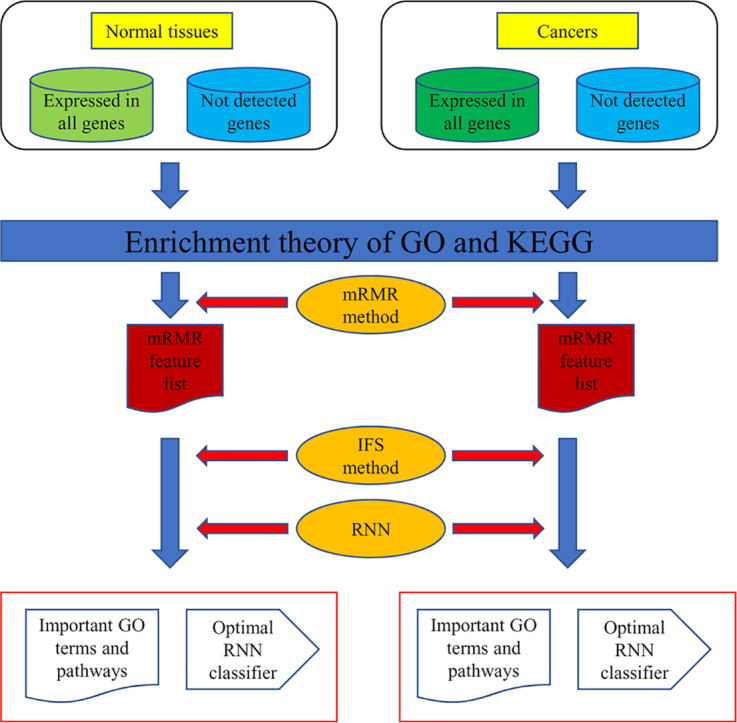

## 1 Introduction

Cancer, which is a general term for describing malignant proliferative diseases with abnormal cell growth, invasion, and metastasis, has been widely confirmed to be one of the major threats to human health [1,2]. Statistics provided by *Lancet* publications [[3], [4], [5]] indicate that over 90 million and approximately 9 million people suffered from and died of cancer, respectively, in 2015 with an average five-year survival rate of approximately 60%. Moreover, epidemiologic statistics indicates that cancer has been regarded as one of the leading killers of humans, ranking just behind infectious and cardiovascular and cerebrovascular diseases, thereby seriously threatening human health [6,7]. However, the basic pathogenic characteristics and underlying mechanisms of cancer, even on the tissue level, have yet to be completely revealed and remains to be explained by further studies.

Gene expression analysis reflects the quantity and quality of messenger RNAs in certain cell subtypes and has its tissue specificity [8]. As a core intermediate segment of the so-called*central dogma*, gene expression profile can relatively represent and describe the detailed biological status and related biological functions [9]. Therefore, gene expression analysis/profiling has long been regarded as an effective parameter for measuring and describing the characteristics of certain biological processes in specific tissue subtypes. In oncology studies, the identification and validation of tumor-specific biological processes is a major approach to revealing crucial carcinogenic factors and processes. Recent publications [[10], [11], [12]] have indicated that the expression profiles of tumor and normal tissues are relatively different, thereby signifying their distinctive biological metabolism processes [12]. Therefore, gene expression profile function analysis may be a relatively effective method for identifying potential tumor-specific pathogenic factors and processes.

However, the traditional identification of gene expression panorama profile is difficult and expensive; hence, functional enrichment analyses of the distinctive expression genes is impossible to perform on the entire transcriptome level [13]. The traditional gene expression analysis has two major limitations: (1)focus on a few limited functional genes and not on the entire transcriptome level and (2)concentrates on the biological function of each differential expressed gene and not on their functional enrichment. Given the development of high throughput sequencing technologies, transcriptome analyses are deemed to be an economical and effective high throughput sequencing based approach to identify tissue specific gene expression patterns on the entire transcriptome level and reveal the detailed expression characteristics of each tissue subtype, thereby addressing the first limitation [14]. Various studies [[15], [16], [17]] have introduced transcriptome sequencing and analysis into oncology studies and revealed the distinctive expression pattern of tumor and normal tissues in different tumor subtypes. However, the so-called expression pattern identification can only reveal the distinctive expression genes (i.e., genes with high or low expression level) but not their related biological functions. To address the second limitation, we introduced two bioinformatics concepts to summarize the function enrichment of genes with different expression levels: gene ontology (GO) [18] and Kyoto Encyclopedia of Genes and Genomes (KEGG) pathways [19]. These pathways have been extensively reported and applied to describe the biological functions and certain cellular components of a few screened gene clusters [20,21], thereby providing an accurate reference for biological functions, annotating the differential gene expression distribution, and improving the level of transcriptomic function analysis from a single gene to gene clusters.

A study [22] has recently revealed the differential expression pattern of 32 tumor and normal tissues. However, the aforementioned study remained limited to the gene level and did not identify the optimal biological processes, in which genes may be enriched. These biological processes can distinguish genes of different clusters with a differential expression level. The current study further extended the aforementioned research. We used the transcriptomic data provided by the preceding study [22] as basis to simply distinguish the genes with a specific expression pattern into two subgroups: (1) expressed in all (detected in all 32 tissues/cancers with FPKM >1) and (2) not detected (FPKM <1 in all tissues/cancers). For convenience, these two gene types are called widely and rarely expressed genes, respectively. Thereafter, we applied functional enrichment analysis on the two subgroups of genes rather than perform the classification on the gene level. First, each investigated gene was encoded into a vector via enrichment theory of GO and KEGG. Second, the minimum redundancy maximum relevance (mRMR) [23], which is a well-known feature selection method, was employed to extensively analyze the GO and KEGG features, thereby producing the mRMR feature list. Third, the incremental feature selection (IFS) [24], which uses recurrent neural network (RNN) [25] as the classification algorithm, was applied to this feature list. Accordingly, the optimal enriched GO terms, which describe either biological processes, cellular components, or molecular functions; and KEGG pathways for the distinction of the two groups of genes in the cancer and normal tissues, are extracted individually. Lastly, we compared the obtained GO terms and KEGG pathways of the cancer and normal tissues, thereby revealing the tumor-specific enrichment items. On one hand, this study may identify the specific biological processes that can distinguish genes with a distinctive expression pattern (FPKM <1 or > 1 in all samples) in multiple tissue subtypes, thereby raising the gene expression distribution analysis to the functional level. On the other hand, the comparison of the screened biological processes in the tumor and normal tissues may reveal the tissue specificity and carcinogenic contribution of the differentially expressed genes' function distribution.

## 2 Materials and Methods

### 2.1 Datasets

We accessed original materials from Uhlen et al.'s study [22] (Table S2), in which 19,571 genes were categorized into several clusters in normal tissues or cancers. The current study aims to investigate the genes that were expressed in all tissues/cancers (FPKM >1 in all samples) or not detected (FPKM <1 in all samples). The genes that were expressed in all tissues/cancers represent the widely existing common functions, while the genes that were not detected represent the rarely expressed genes.

**Fig. 1:**
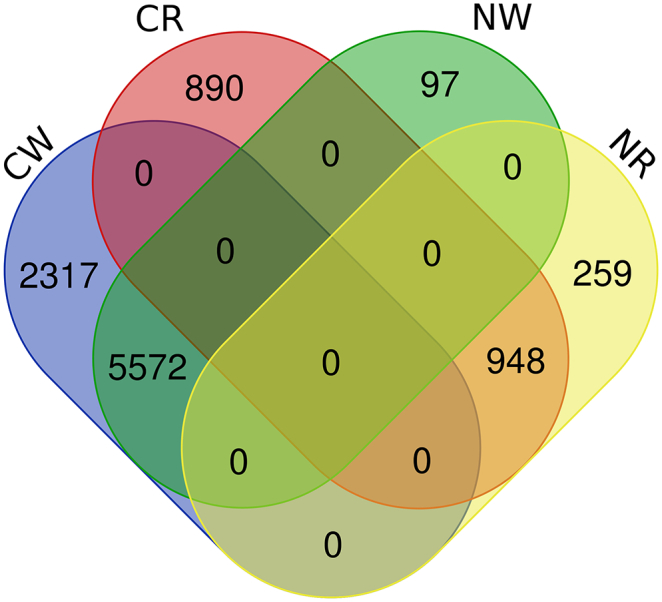
A Venn diagram to illustrate the widely and rarely expressed genes for normal tissues and cancers. NW represents the set consisting of widely expressed genes for normal tissues, NR represents the set consisting of rarely expressed genes for normal tissues, CW represents the set consisting of widely expressed genes for cancers, CR represents the set consisting of rarely expressed genes for cancers.

From normal tissues, we extracted 5873 widely expressed genes and 1810 rarely expressed genes. From cancers, we extracted 8173 widely expressed genes and 2570 rarely expressed genes. Each investigated gene in this study was encoded via enrichment theory of GO and KEGG [26]. Thus, the genes with unavailable enrichment information were discarded. Lastly, we obtained 5669 widely expressed genes and 1207 rarely expressed genes from normal tissues and 7889 widely expressed genes and 1838 rarely expressed genes from cancers. To clearly illustrate the distribution of above-mentioned widely and rarely expressed genes, a Venn diagram was plotted in Fig. 1, from which we can see that lots of widely expressed genes for normal tissues are also widely expressed genes for cancers and vice versa, rarely expressed genes also have such property.

The genes were categorized into two subgroups in the normal tissues or cancers. To describe the differences between the genes in these two clusters, we set up a binary classification problem for normal tissues and cancers, respectively. For convenience, widely expressed genes were deemed as positive samples, while rarely expressed genes were called negative samples.

### 2.2 Feature Construction

This study aims to perform functional enrichment analysis on the two clusters of genes in the normal tissues and cancers. Accordingly, we employed the GO terms and KEGG pathways to quantify the functions of genes. Enrichment theory [26] of GO and KEGG was adopted to encode each gene. Compared with the classic encoding method that always uses 0 or 1 to represent whether a gene is annotated by a GO term or pathway, the encoding method used in this study can produce features with less sensitivity. The obtained features are more robust [27] because enrichment theory can consider the significance of the overlap between a set about the gene and another set about the GO term or pathway. The detailed descriptions of how to encode each gene via such method are presented as follows.

#### 2.2.1 GO Enrichment

Given gene *g* and the GO term *G*_*j*_, the gene set *GSG*_*j*_ contains the annotated genes of *G*_*j*_ and gene set *GS*(*g*) containing the interacting genes of *g* are defined and can be accessed using the protein–protein interaction (PPI) network reported in STRING [28]. The GO enrichment score between *g* and *G*_*j*_ is the hypergeometric test *P* value of *GS*(*g*) and *GSG*_*j*_, which is calculated as follows:

(1) $$S_{\mathrm{GO}}\left(g,G_{j}\right)=-\log_{10}\left(\sum_{k=m}^{n}\frac{\left(\begin{array}{c} M\\ k \end{array}\right)\;\left(\begin{array}{c} N-M\\ n-k \end{array}\right)}{\left(\begin{array}{c} N\\ n \end{array}\right)}\right)$$

where *N* represents the total number of human genes, *M* is the number of genes in *GSG*_*j*_, *n* the number of genes in *GS*(*g*), and *m* the number of genes in the intersection of *GSG*_*j*_ and *GS*(*g*). The high outcome of Eq. (1) means that *g* is highly enriched on *G*_*j*_. A total of 20,681 GO terms were used in this study, thereby resulting in 20,681 GO enrichment scores for each gene.

#### 2.2.2 KEGG Enrichment

The definition of KEGG enrichment score is similar to that of GO enrichment score. For a given gene *g* and one KEGG pathway *P*_*j*_, *GS*(*g*) is the same as that in the GO enrichment and *GSP*_*j*_ is the gene set containing the annotated genes of *P*_*j*_. The KEGG enrichment score between *g* and *P*_*j*_ is the hypergeometric test *P* value of *GS*(*g*) and *GSP*_*j*_, which is calculated as follows:

(2) $$S_{\text{KEGG}}\left(g,P_{j}\right)=-\log_{10}\left(\sum_{k=m}^{n}\frac{\left(\begin{array}{c} M\\ k \end{array}\right)\;\left(\begin{array}{c} N-M\\ n-k \end{array}\right)}{\left(\begin{array}{c} N\\ n \end{array}\right)}\right)$$

where *N* and *n* are identical to those in Eq. (2), *M* is the number of genes in *GSP*_*j*_, and *m* is the number of genes in the intersection of *GSP*_*j*_ and *GS*(*g*). Similarly, a high score indicates that *g* is highly enriched on *P*_*j*_. A total of 297 KEGG pathways were adopted in this study, thereby producing 297 KEGG enrichment scores to represent the relationships between each gene and the 297 pathways.

By collecting all the GO and KEGG enrichment scores, any investigated gene can be represented by a 20,978- dimensional vector. Several GO and KEGG features can be extracted by applying advanced computational methods on all the gene vectors. The corresponding GO terms and KEGG pathways can be obtained, which may be important for the distinction of the two clusters of genes in normal tissues and cancers.

### 2.3 Feature Selection Method

Several feature selection methods are necessary to analyze the 20,978 GO or KEGG features. Hence, we designed a two-stage feature selection to extract important features, thereby obtaining the important GO terms and KEGG pathways. In the first stage, the mRMR [23] method was adopted to analyze all features, thereby resulting in a feature list. Thereafter, we applied IFS [24] with a supervised classifier RNN [25] to the feature list in the second stage, thereby selecting discriminate features for classifying the genes in two clusters.

#### 2.3.1 Minimum Redundancy Maximum Relevance (mRMR)

In the field of machine learning, several feature selection methods have been proposed to deal with different types of data, such as ReliefF [29], maximum relevance maximum distance (MRMD) [30], etc. Different methods have their own advantages. Here, we selected the mRMR method, proposed by Peng et al. [23], because it is a widely used feature selection method and deemed an excellent method for analyzing the importance of features. To date, it has been applied in several biological problems [[31], [32], [33], [34], [35], [36], [37], [38], [39], [40]].

The mRMR method adopts mutual information (MI) to indicate the relationships between two variables. For two variables *x* and *y*, the MI can be calculated as follows:

(3) $$I\left(x,y\right)=\iint p\left(x,y\right)\log\frac{p\left(x,y\right)}{p\left(x\right)p\left(y\right)}\mathrm{dxdy}$$

where *p*(*x*) and *p*(*y*) indicate the marginal probabilistic densities of *x* and *y*, respectively, and *p*(*x*,*y*) represents the joint probabilistic density of *x* and *y*. mRMR generates the mRMR feature list that indicates the importance of each feature. This list is produced in terms of two criteria: (1) Max-Relevance between features and targets and (2) Min-Redundancy between features. These two criteria are quantified using MI. Let Ω be the set containing all features and Ω_*s*_ be the set comprising the features that have already been selected. For each unselected feature *f*, evaluate its relevance to target variable *c* using *D* = *I*(*c*, *f*) and further measure its redundancies to already selected features in Ω_*s*_ by $R=\frac{1}{\left|\Omega_{s}\right|}\sum_{f'\in \Omega_{s}}I\left(f,f^{'}\right)$ (If Ω_*s*_ is empty, *R* is set to zero). The next selected feature is the feature that has Max-Relevance to the targets and Min-Redundancy to the already selected features in Ω_*s*_. Thus, a feature with maximum *D*-*R* value is selected and put it into Ω_*s*_. After all features have been selected, that is, Ω_*s*_ = Ω, each feature is assigned a selection order based on which the mRMR feature list is produced. That is, the first selected feature is at the top rank, the second selected feature is second, and so on. The obtained mRMR feature list (*F*) is formulated as follows:

(4) $$F=\left[f_{1},f_{2},\ldots ,f_{N}\right]$$

where *N* is the total number of features (*N* = 20,978 in this study). Evidently, the features with high ranks in *F* are relatively important.

#### 2.3.2 Incremental Feature Selection (IFS)

Determining which features are of immense importance based only on *F* is relatively difficult. Accordingly, the IFS method and RNN were employed. However, it was impossible to test all possible feature sets by the original IFS procedures because there were >20,000 sets that should be tested for genes of normal tissues or cancers, respectively. To save time, we performed a two-step IFS method. In the first step, we generated a series of feature subsets with step ten from feature list *F*, formulated as S_1_^1^, S_2_^1^, …, S_*M*_^1^, where S_i_^1^ = [f_1_, f_2_, …f_i∗10_]. That is, the first 10☓*i* features in *F* constitute the *i*th subset. For these feature subsets, a classification algorithm RNN was built on the samples represented by the features from each feature subset. We also evaluated the corresponding performance of RNN using a 10-fold cross-validation [41]. The feature set that can provide RNN with the best classification performance can be accessed. According to the number of features in the above feature set, a small interval around such number was determined. In the second step, we constructed all possible feature sets, in which the numbers of features were in the obtained interval. Likewise, a classification algorithm RNN was built on samples represented by features in each of above sets and evaluated its performance via 10-fold cross-validation. Accordingly, a feature set yielding the best performance for RNN can be obtained. The features in this set are called optimum features, while the corresponding RNN classifier is termed as the optimum classifier.

### 2.4 Recurrent Neural Network (RNN)

We need a prediction engine to classify the genes in two groups for normal tissues and cancers. The current study employed RNN [25]. A traditional neural network constantly supposes that all inputs (and outputs) are independent of each other. However, the output for sequential data is related to previous computations. RNN is a type of neural network with loops inside, thereby enabling information to persist for the subsequential outputs. RNNs can theoretically memorize information with any long sequences. However, they are limited in practice to looking back only a few steps.

Long short term memory (LSTM) network [25] is a special type of RNN that can learn long-term dependencies [42]. LSTM includes three types of layers: (1) forget gate layer, (2) input gate layer, and (3) output layer. First, a forget gate layer is used to decide which information of the previous layer should be disregarded. Second, an input gate layer identifies which information should be passed to the subsequent layer and updates the current state value. Third, an output gate layer decides what parts of the state value can be outputted.

If we assume that we have a sequence {x}^T^, while LSTM has hidden states {h}^T^, cell state {c}^T^, and output {o}^T^, then the preceding steps can be formatted as follows:

(5) $$\begin{array}{l} f_{t}=\text{Sigmoid}\left(W_{f}x_{t}+U_{f}h_{t-1}+b_{f}\right)\\ i_{t}=\text{Sigmoid}\left(W_{i}x_{t}+U_{i}h_{t-1}+b_{i}\right)\\ c_{t}=f_{t}⊙c_{t-1}+i_{t}⊙\tanh\left(W_{c}x_{t}+U_{c}h_{t-1}+b_{c}\right)\\ o_{t}=\text{Sigmoid}\left(W_{o}x_{t}+U_{o}h_{t-1}+b_{o}\right)\\ h_{t}=o_{t}⊙\tanh\left(h_{t}\right) \end{array}$$

where ⊙ is the element-wise multiplication; W_∗_, U_∗_, and b_∗_ are the parameters of LSTM; and i_t_, f_t_, c_t_, and o_t_ are the input, forget, cell, and output gate, respectively.

This study implemented RNN to classify the genes in two groups using Tensorflow [43].

### 2.5 Performance Measurement

This study performed a 10-fold cross-validation [41] to evaluate each model. This method equally and randomly divides the original data set into 10 parts. The samples in each part are singled out one after the other and tested by the model built on the samples in the other nine parts. Compared with another cross-validation called the Jackknife test [44,45], 10-fold cross-validation needs less time and constantly produces similar results. To date, 10-fold cross-validation has been applied to the evaluation of different constructed models [34,36,37,[46], [47], [48], [49]].

To investigate two clusters of genes in normal tissues and cancers, we set up a binary classification problem for each case. For this type of problem, the predicted results can constantly be counted as true positive (TP), true negative (TN), false negative (FN), and false positive (FP), where TP/TN indicates the number of correctly predicted positive/negative samples, while FN/FP denotes the number of incorrectly predicted positive/negative samples. Accordingly, four measurements, namely, sensitivity (SN), specificity (SP), accuracy (ACC), Matthew's correlation coefficient (MCC) [[32], [33], [34],^36^,^37^,^44^,^47^,^50^,^51^] and Youden's index (J) [52] can be calculated. These measurements are defined as follows:

(6) $$\mathrm{SN}=\frac{\mathrm{TP}}{\mathrm{TP}+\mathrm{FN}},$$

(7) $$\mathrm{SP}=\frac{\mathrm{TN}}{\mathrm{TN}+\mathrm{FP}},$$

(8) $$\mathrm{ACC}=\frac{\mathrm{TP}+\mathrm{TN}}{\mathrm{TP}+\mathrm{TN}+\mathrm{FP}+\mathrm{FN}},$$

(9) $$\mathrm{MCC}=\frac{\mathrm{TP}\times \mathrm{TN}-\mathrm{FP}\times \mathrm{FN}}{\sqrt{\left(\mathrm{TP}+\mathrm{FP}\right)\left(\mathrm{TP}+\mathrm{FN}\right)\left(\mathrm{TN}+\mathrm{FP}\right)\left(\mathrm{TN}+\mathrm{FN}\right)}}.$$

(10) J=SN+SP−1

ACC, MCC and Youden's index can evaluate the overall performance of the classifier. In addition, the sizes of the two clusters of genes for normal tissues and cancers had substantial difference. That is, the two constructed datasets were imbalanced. In this case, ACC is not a proper measurement because it does not consider the different sizes of classes, while MCC and Youden's index further consider this fact. According to Section 2.1, widely expressed genes for normal tissues were more than four times as many as rarely expressed genes and the same phenomenon also occurred for widely and rarely expressed genes of cancers. Thus, the investigated datasets were quite imbalanced. In this case, Youden's index is deemed to be a more proper measurement as suggested in some previous studies [[53], [54], [55]]. Thus, we used Youden's index as the key measurement in evaluating the performance of different models and provided other measurements as references.

## 3 Results

**Fig. 2:**
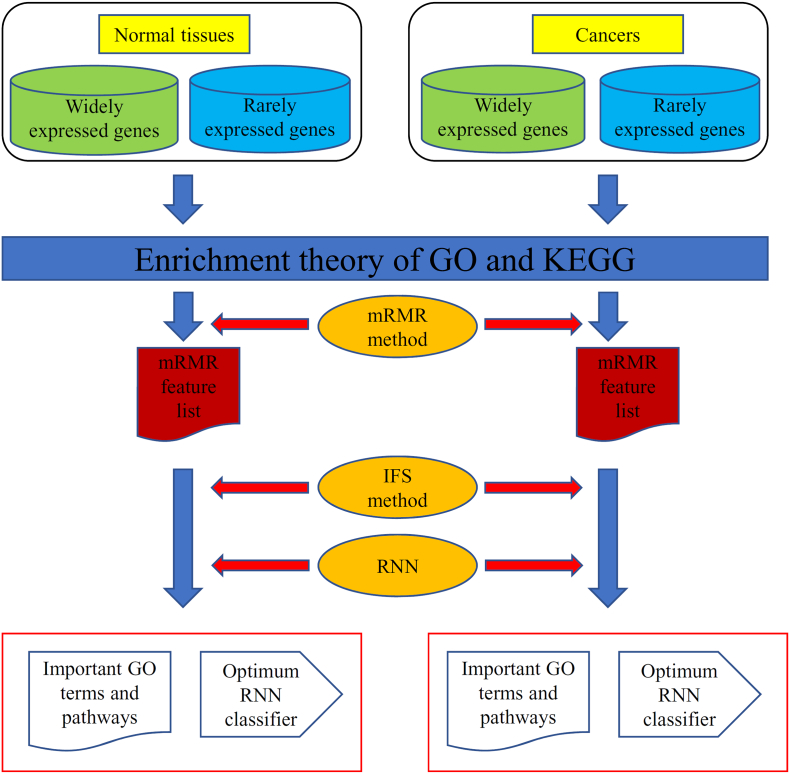
Detailed procedure for investigating the widely and rarely expressed genes of normal tissues and cancers. All investigated genes were encoded using enrichment theory of GO and KEGG. Thereafter, the minimum redundancy maximum relevance (mRMR) method was adopted to analyze the encoded features, thereby resulting in an mRMR feature lists for normal tissues and cancers, respectively. Lastly, the incremental feature selection (IFS) method and recurrent neural network (RNN) were both used to extract the important GO terms and KEGG pathways and construct the optimum RNN classifiers based on the mRMR feature list.

This study investigated two clusters of genes (i.e., widely and rarely expressed genes) in normal tissues and cancers using the GO terms and KEGG pathways. Several advanced computational methods were incorporated in this study. Fig. 2 illustrates the entire procedure.

### 3.1 Results of the mRMR Method

The 5669 widely expressed genes and 1207 rarely expressed genes of normal tissues were represented by 20,978 GO or KEGG features (see description in Section 2.2). A powerful feature selection method (i.e., mRMR) was applied to analyze these features, thereby generating an mRMR feature list (provided in Supplementary Material S1).

The 7889 widely expressed genes and 1838 rarely expressed genes of cancers were processed in the same manner. We also obtained an mRMR feature list (provided in Supplementary Material S2).

### 3.2 Results of the IFS Method and RNN

A two-step IFS method was employed to extract the discriminative GO and KEGG features for widely and rarely expressed genes of normal tissues and cancers. In the first step, we used step 10 to construct a series of feature sets. That is, we constructed feature sets that contain first 10, 20, 30, and so on features in the mRMR feature set. Thereafter, a powerful classification algorithm (i.e., RNN) was adopted as the prediction engine in evaluating these feature sets. For each feature set, all investigated genes (widely and rarely expressed genes) of the normal tissues and cancers were represented by the features in the set, while an RNN classifier was executed on this representation. 10-fold cross-validation was used to evaluate the performance of this RNN classifier. The results were counted as SN, SP, ACC, MCC and Youden's index using Eqs. (6), (7), (8), (9), (10). Furthermore, according to the obtained measurements, we determined a small interval for the second step of IFS procedures. All possible feature sets, whose sizes were in this interval, were constructed and evaluated by RNN via 10-fold cross-validation. The results were also counted as measurements listed in Eqs. (6), (7), (8), (9), (10).

**Fig. 3:**
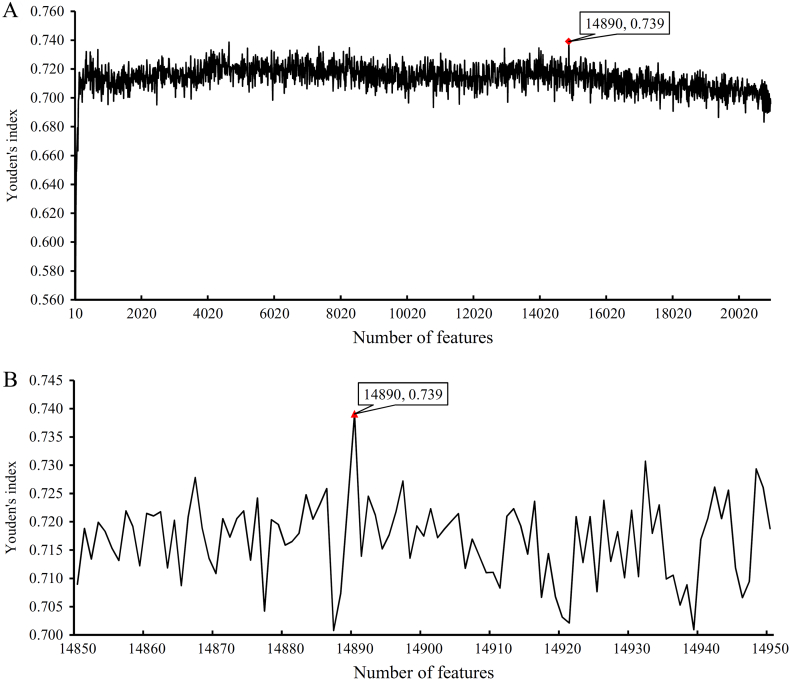
IFS curves to show the trends of Youden's indexes that correspond to the number of features involved in constructing the recurrent neural network (RNN) classifier for normal tissues. (A) IFS curve with step 10. (B) IFS curve between 14,850 and 14,950 with step 1.

For normal tissues, the obtained SNs, SPs, ACCs, MCCs and Youden's indexes yielded by RNN on different feature sets that were constructed in the first and second steps of IFS method are provided in Supplementary Material S3. Youden's index was selected as the major measurement. For the Youden's indexes obtained in the first step of IFS method, we plotted an IFS curve using Youden's index as the Y-axis and the number of features in the set as the X-axis (see Fig. 3(A)). It can be observed that this curve first follows a sharp increasing trend and stabilizes thereafter. The highest Youden's index was 0.739 when the first 14,890 features were used. Thus, we determined the interval [14,850, 14,950] for the second step of IFS method. Likewise, for ease of observation, an IFS curve was also plotted, as shown in Fig. 3(B), from which we can see that the highest Youden's index was still 0.739 and it was still obtained by the first 14,890 features. Therefore, we confirmed that these 14,890 features were the optimum features for RNN and the corresponding RNN classifier was the optimum RNN classifier for detecting the widely and rarely expressed genes of the normal tissues. Table 1 lists the detailed performance of this classifier. SN, SP, ACC and MCC were 0.965, 0.774, 0.932, and 0.758, respectively. SP was substantially lower than the SN because the number of rarely expressed genes (termed as negative samples) were less than that of the widely expressed genes (termed as positive samples).

**Table 1:** Performance of the optimum RNN and RF classifiers in detecting the widely and rarely expressed genes of normal tissues.

Prediction engine	Number of features	SN	SP	ACC	MCC	Youden's index
RNN	14,890	0.965	**0.774**	0.932	**0.758**	**0.739**
RF	372	**0.985**	0.696	**0.934**	**0.758**	0.681

**Fig. 4:**
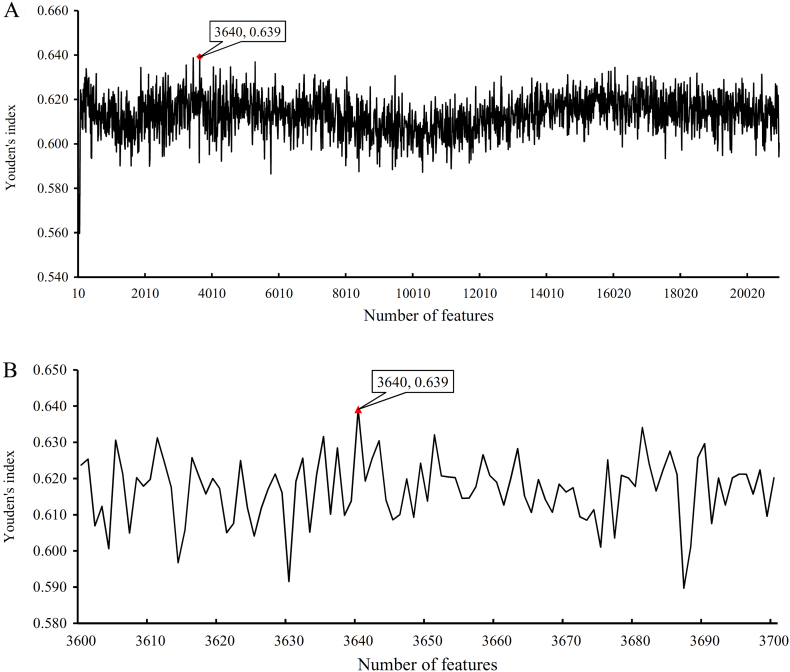
IFS curves to show the trends of Youden's indexes that correspond to the number of features involved in constructing the recurrent neural network (RNN) classifier for cancers. (A) IFS curve with step 10. (B) IFS curve between 3600 and 3700 with step 1.

We performed the same procedure for cancers. The obtained SNs, SPs, ACCs, MCCs, Youden's indexes are listed in Supplementary Material S4. For the Youden's indexes yielded in the first step of IFS method, we also plotted an IFS curve to describe the performance of RNN on different feature sets (see Fig. 4(A)). The highest Youden's index was 0.639 when the first 3640 features were used. Accordingly, an interval [3600, 3700] was determined and tested in the second step of IFS method. An IFS curve was also plotted, as shown in Fig. 4(B), to illustrate the obtained Youden's indexes. We can see that the highest Youden's index was still 0.639 and it was still yielded by the first 3640 features. Accordingly, these 3640 features were termed as the optimum features for RNN, while the RNN classifier based on these features was called the optimum RNN classifier for distinguishing the widely and rarely expressed genes in cancers. Table 2 lists the detailed performance of this classifier. SN, SP, ACC, and MCC were 0.947, 0.693, 0.899, and 0.660 respectively. In addition, SP was substantially lower than SN because of the considerable difference between the numbers of the widely and rarely expressed genes.

**Table 2:** Performance of the optimum RNN and RF classifiers in detecting the widely and rarely expressed genes of cancers.

Prediction engine	Number of features	SN	SP	ACC	MCC	Youden's index
RNN	3640	0.947	**0.693**	0.899	0.660	**0.639**
RF	80	**0.970**	0.624	**0.905**	**0.666**	0.594

### 3.3 Comparisons of the IFS Method and Random Forest

This study adopted RNN as the prediction engine in evaluating the discriminative ability of the different feature sets and constructing the optimum classifier. To show its reasonability, we further employed another popular machine learning algorithm (i.e., random forest (RF)) [56] following the same procedures for comparison. This algorithm has been applied to the construction of several effective prediction models that deal with different biological problems [36,[57], [58], [59], [60], [61]].

**Fig. 5:**
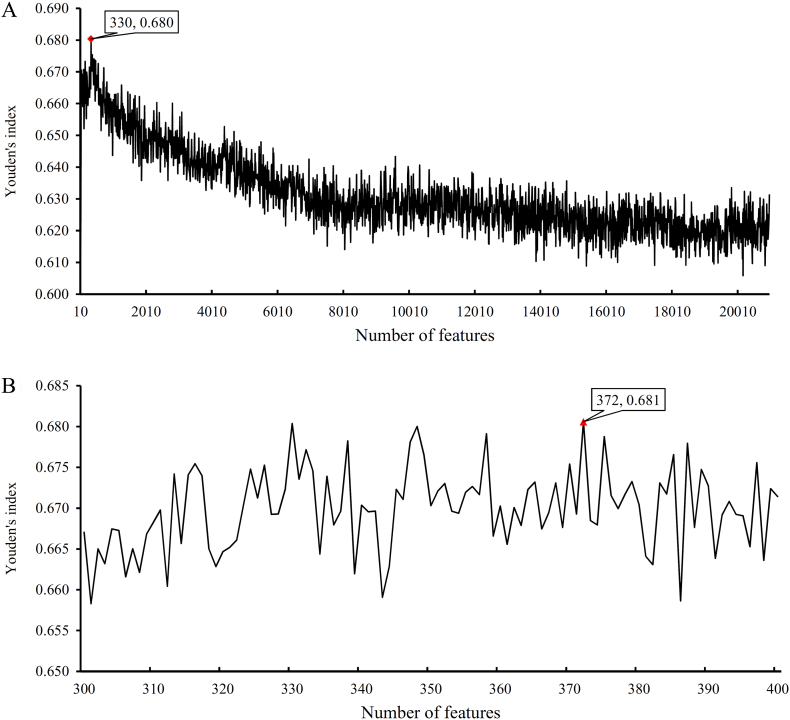
IFS curves to show the trends of Youden's indexes that correspond to the number of features involved in constructing the random forest (RF) classifier for normal tissues. (A) IFS curve with step 10. (B) IFS curve between 300 and 400 with step 1.

**Fig. 6:**
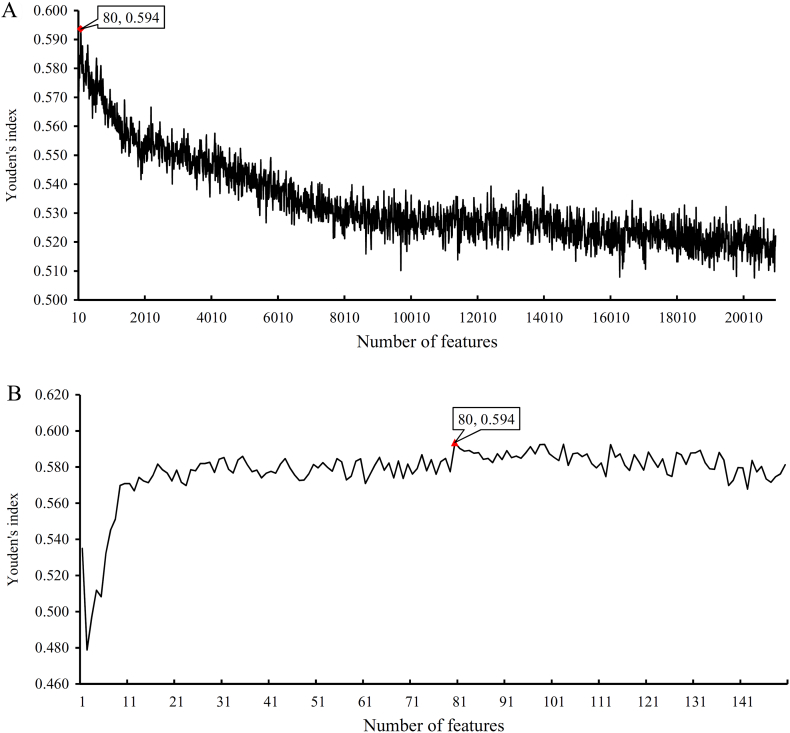
IFS curves to show the trends of Youden's indexes that correspond to the number of features involved in constructing the random forest (RF) classifier for cancers. (A) IFS curve with step 10. (B) IFS curve between 1 and 150 with step 1.

For normal tissues and cancers, the performance of RF on the different constructed feature sets is provided in Supplementary Materials S5 and S6, respectively. Fig. 5, Fig. 6 present the IFS curves that show the relationships between Youden's index and the number of features used. For normal tissues, the highest Youden's index was 0.680 in the first step of IFS method when the first 330 features in the mRMR feature list were used (see Fig. 5(A)). Then, we further evaluated the feature sets in interval [300, 400] (see Fig. 5(B)), from which we can see that the highest Youden's index was 0.681 and it was yielded by the first 372 features in the list. Therefore, we built an optimum RF classifier for normal tissues by using these 372 features to represent widely and rarely expressed genes. Table 1 shows the detailed performance of such optimum RF classifier. For cancers, in the first step of IFS method, the highest Youden's index was 0.594 when the first 80 features were used (see Fig. 6(A)). Then, an interval [1, 150] was determined and all feature sets in this interval were evaluated by RF, resulting an IFS curve, as shown in Fig. 6(B). It can be observed that the highest Youden's index was 0.594 when the first 80 features were used. Accordingly, an optimum RF classifier using these features was built. The detailed performance of this classifier is listed in Table 2.

For normal tissues, the optimum RF classifier yielded an SN of 0.985, SP of 0.696, ACC of 0.934, MCC of 0.758, Youden's index of 0.681 (see row 3 of Table 1). The optimum RF classifier produced a higher SN, lower SP, higher ACC, equal MCC compared with those of optimum RNN classifier. However, the optimum RNN classifier yielded a higher Youden's index, thereby indicating that the RNN classifier was superior to the RF classifier. For cancers, the optimum RF classifier yielded a higher SN, lower SP, higher ACC, and higher MCC. However, RF still produced a lower Youden's index than that of optimum RNN classifier. Thus, the optimum RNN classifier for caners was still better than the optimum RF classifier. These results suggest that RNN was a good choice for distinguishing widely and rarely expressed genes.

## 4 Discussion

**Fig. 7:**
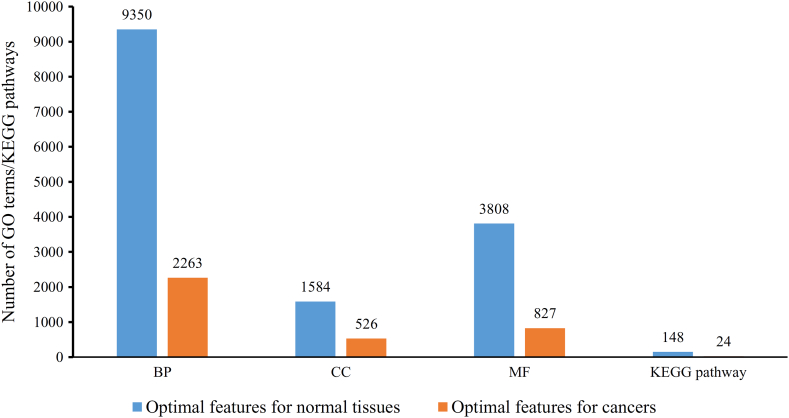
The distribution of optimum features used in the optimum recurrent neural network (RNN) classifiers for classifying widely and rarely expressed genes. BP represents biological process, CC cellular component and MF molecular function.

In this study, the GO terms and KEGG pathways were introduced for the first time to describe the differential gene expression pattern distribution at the functional level and not just at the gene level as in previous studies. We screened out several GO terms and KEGG pathways that can distinguish widely expressed genes (FPKM >1 in all samples) and rarely expressed genes (FPKM <1 in all samples) for normal tissues and cancers. For normal tissues, the optimum RNN classifier used 14,890 features, which involved 14,742 GO terms and 148 KEGG pathways. The optimum RNN classifier for cancers adopted 3640 features, corresponding to 3616 GO terms and 24 KEGG pathways. The distribution of above optimum features for normal tissues and cancers is illustrated in Fig. 7. It can be observed that the biological process GO terms were most, followed by molecular function GO terms, cellular component GO terms and KEGG pathways. This section discussed the investigation on several top GO terms and KEGG pathways. Furthermore, the enriched KEGG and GO terms in tumor tissues are relatively different from those in normal tissues, thereby reflecting the potential specific biological characteristics of tumors. The detailed analyses of each predicted KEGG and GO terms are presented as follows.

### 4.1 Analysis of the GO Terms and KEGG Pathways that Can Distinguish the Widely and Rarely Expressed Genes of Normal Tissues

We obtained the mRMR feature list that indicated the importance of the GO terms and KEGG pathways. Hence, we selected several GO terms with top ranks from the mRMR feature list for detailed analysis.

**GO:0010992** (ubiquitin homeostasis) was the top distinguisher for widely and rarely expressed genes of normal tissues. A recent publication presented by the University of Ghent indicates that genes that contribute to ubiquitin homeostasis turns out to be up-regulated in normal homeostatic epithelial tissues, thereby activating the NF-kB regulatory pathways. Therefore, the genes related to this GO term can maintain a relatively high expression level in certain normal tissues compared with genes that contribute to other biological processes [62]. The following GO term, **GO:0071875**, describes the adrenergic receptor signaling pathway. As a specific G protein-coupled receptor, adrenergic receptor has been identified in multiple tissues with a specific expression level [63,64]. Therefore, such biological process, which describes the biological function of adrenergic receptor, can be confirmed to be one of the effective biological processes that distinguish widely and rarely expressed genes. The following GO term was a specific mRNA expression regulatory biological process, **GO:0035925**, that describes the mRNA 3’-UTR AU-rich region binding. Undoubtedly, the biological process can distinguish the widely or rarely expressed genes of normal tissues. Similarly, the downstream of the gene expression, the synthesis of functional proteins associated biological processes, such as **GO:0060904**, that describes the regulation of protein folding in endoplasmic reticulum was identified as a potential distinguisher for widely and rarely expressed genes. Given the correspondence of gene expression and protein synthesis, such biological process is another potential marker for gene expression in normal tissues. The next GO term, **GO:0072186**, describes metanephric cap morphogenesis. The genes that contribute to such biological process have been confirmed to have a quite high expression pattern in the normal tissue of metanephric cap in early kidney development during the embryonic phase [65].

GO term **GO:0007600**, which describes the sensory perception, was also identified as an optimal parameter for the distinction of genes with a high or low expression pattern. Recent publications have indicated that the expression level of sensory perception in normal tissues, such as skin, has been confirmed to be relatively high (FPKM >1) compared with other non-relevant genes [66]. For organs that have nothing to do with the senses, the expression pattern of genes enriched in such a biological process may be quite low (FPKM <1). The next GO term, **GO:0044444**, describes a specific cellular component (i.e., cytoplasmic part). Given that the GO cellular component annotation of genes describes the subcellular distribution of certain gene or gene products, such enrichment indicates that gene products located or not located at the cytoplasmic part have a distinctive expression pattern in normal tissues [67,68]. The majority of the gene products spread over the cytoplasmic part. Therefore, genes enriched in such a cellular component may have a higher expression pattern than other genes. **GO:0071880** describes the adenylate cyclase-activating adrenergic receptor signaling pathway. Genes that contribute to or can be enriched in such biological processes turn out to have a relatively high tissue specificity. In pineal, a small endocrine gland in the center of the brain, the expression level of functional genes enriched in such a GO term has been confirmed to be higher than those in other tissues (FPKM >1) [69,70].

The following GO term, **GO:0005882**, describes a cellular component. This GO term describes the intermediate filament, which is a functional major component of mitosis. In terminative cell subtypes, the expressed genes enriched in such a GO term have been confirmed to be down-regulated [71]. Furthermore, in proliferative cell/tissue types, genes enriched in such a biological process turns out to be up-regulated [71,72]. Similarly, GO terms, such as **GO:0050877** and **GO:0090095**, were also identified to contribute to the recognition of differential gene expression pattern. The biological functions of each detailed gene ontology and KEGG pathways cannot be analyzed in detail due to the length limitation of this manuscript. According to recent publications, GO:0050877 and GO:0090095 can be confirmed to describe the differential expression pattern in normal cells [73].

### 4.2 Analysis of the GO Terms and KEGG Pathways that Can Distinguish the Widely and Rarely Expressed Genes of Cancers

We also obtained a few important GO terms and KEGG pathways for the distinction of the widely and rarely expressed genes of cancers in terms of the mRMR feature list provided in Supplementary Material S2. Moreover, we analyzed a few important ones.

**GO:0010992**, as the top GO term for cancers, describes ubiquitin homeostasis. Recent publications [[74], [75], [76]] have indicated that the homeostasis of ubiquitin is inhibited during tumorigenesis, while the genes that contribute to its maintenance has been reported to be down-regulated. These results indicated that a few low expressed genes may be enriched in this GO term, but no highly expressed genes can be enriched in such a biological process. Apart from GO:0010992, another GO term (**GO:0007600**) was also identified to contribute to the distinction of genes with a differential expression level in cancers. Describing sensory perception, this GO term describes the biological process required for an organism to receive and recognize a sensory stimulus. For the distinctive role of such a biological process, considering cancer pain, which is related to sensory perception, turns out to be one of the major challenge in cancer treatment [77,78]. A few specific sensory perception associated genes are intentionally up- or down-regulated in tumor [79], thereby inducing the functional enrichment distinction of genes with a high or low expression level. The third GO term, **GO:0035925**, describes a specific molecular function (3’ UTR AU-rich region binding), which has also been confirmed to be functionally related to tumorigenesis [80,81]. For the potential distinctive function of this GO term, given that the binding capacity of genes (such as SOD1, HuR, and EGR1) [[81], [82], [83]] in tumor on the 3’UTR AU-rich region is directly associated with its transcriptional and translational levels, such a molecular function can be identified as a potential parameter for the distinction of genes with different expression levels.

The next GO term describes a quite significant biological process, the regulation of metanephric cap mesenchymal cell proliferation (**GO:0090095**). As a tissue specific biological process, the involvement of this GO term in various tumor associated genes, such a cadherin family, p38, and MYC, has been confirmed [84,85] In cancers, particularly renal carcinoma, genes that contribute to GO:0090095 have been reported to be up-regulated compared with other irrelevant genes [86], thereby conforming to the expression profile clustering function of such a GO term. **GO:0004872**, which describes the general receptor activity, may have also been enriched by genes with high or low expression patterns. Recent publications have indicated that the expression level of the receptor biological function-associated genes during tumorigenesis is relatively different from that of other functional genes. In the ER+ or HER2+ breast cancer, the expression level of the estrogen and human epidermal growth factor receptors turn out to be quite higher than that of other compared genes [[87], [88], [89], [90]].

Apart from the GO terms, a specific KEGG pathway, neuroactive ligand-receptor interaction (**hsa04080**), was identified to distinguish genes with a high or low expression pattern in cancers. In early 2010, a specific clinical study on liver tissues (cancer and hepatitis) has confirmed that genes that constitute the neuroactive ligand-receptor interaction associated network may be up-regulated and have a high expression pattern (FPKM >1) in cancers [91]. Furthermore, another optimal pathway, **hsa04740** (olfactory transduction), was identified as a specific expressed pathway that distinguishes the differential expressed genes in cancers. Recent publications have indicated that genes encoding olfactory receptors have been extensively reported to have a specific expression pattern in multiple tumor subtypes, such as melanoma [92] and lung cancer [93]. A specific gene, OR2C3, which contributes to such a biological pathway, has been confirmed to have an abnormally high expression pattern in melanoma [94], thereby confirming the distinctive function of such a pathway in unique subtypes of tumors.

### 4.3 Analysis of the KEGG and GO Terms that Are Differentially Enriched in Cancer and Normal Tissues

**Fig. 8:**
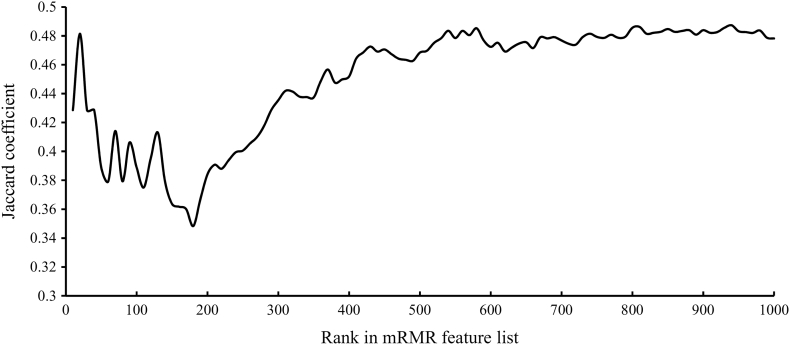
Trend of the Jaccard coefficients that correspond to sets containing the top features in the mRMR feature lists of normal tissues and cancers. The Jaccard coefficients are between 0.3 and 0.5, thereby suggesting that the top features in the mRMR feature lists of normal tissues and cancers have common and different features.

We identified a few effective biological processes shared by normal tissues and cancers and screened out tumor-specific expression patterns described by the GO terms and KEGG pathways. To confirm this result, we counted the Jaccard coefficients of the sets that contain the top features in the mRMR feature list of normal tissues and cancers (see Fig. 8). The Jaccard coefficients were between 0.3 and 0.5, thereby indicating that the top features of the normal tissues and cancers have common and different features.

Several GO terms and KEGG pathways were identified in normal tissues and cancers. **GO:0007600** (sensory perception) has been validated to be capable of distinguishing genes with high or low expression patterns in the tumor and normal tissues. Apart from such a biological process, another GO term, **GO:0090095** (metanephric cap mesenchymal cell proliferation), was also inferred to contribute to gene expression clustering in normal tissues and cancers. The preceding analysis indicates that given such a biological process involves functional tumor associated genes, such as cadherin family, p38, and MYC, we can reasonably speculate that the genes that participate in such a biological process are highly expressed. Meanwhile, genes that participate in the metanephric cap mesenchymal cell proliferation in normal tissues may also be down-regulated with a specific expression pattern that can be distinguished from those of other functional genes.

Apart from such shared GO terms, we also identified some unique tumor specific enriched items, reflecting the unique gene expression pattern in tumor tissues. A specific molecular function (**GO:0035925**) was deemed to be unique in tumor tissues. GO:0035925 describes a specific molecular function named 3’ UTR AU-rich region binding. Given that 3’ UTR AU-rich has a unique expression pattern (FPKM >1) in cancers but not in normal tissues, such an item can be regarded as a potential tumor specific biomarker at the transcriptomic level [80,81].

Several top GO terms and KEGG pathways can be confirmed to distinguish widely expressed genes (FPKM >1) and rarely expressed genes (FPKM <1). These identified GO terms and KEGG pathways in tumor or normal tissues can reflect the tumor specific gene expression pattern and its related biological processes. Recent publications have indicated that extracted GO and KEGG terms are functionally related to cell proliferation, abnormal energy metabolism, and transcriptomic regulation, thereby revealing the potential relationship between tissue specific gene expression profiling and biological functions. On the one hand, the findings of this study can reveal the functional distinction of genes with different expression levels. On the other hand, this research contributes to the identification of the core-revealed functional distinction, thereby possibly distinguishing normal tissues and cancers further, while revealing the specific gene expression distribution of tumor tissues.

## Appendix A Supplementary Data

Supplementary Material S1. mRMR feature list generated by the mRMR method on two clusters of genes in normal tissues. Image 1

Supplementary Material S2. mRMR feature list generated by the mRMR method on two clusters of genes in cancers. Image 2

Supplementary Material S3. Performance of the RNN in classifying two clusters of genes in normal tissues under different numbers of features. Image 3

Supplementary Material S4. Performance of the RNN in classifying two clusters of genes in cancers under different numbers of features. Image 4

Supplementary Material S5. Performance of the RF in classifying two clusters of genes in normal tissues under different numbers of features. Image 5

Supplementary Material S6. Performance of the RF in classifying two clusters of genes in cancers under different numbers of features. Image 6
